# Evaluating the Oncologic and Safety Outcomes of High-Dose Palliative Radiation Treatment with 30 Grays in Five Fractions

**DOI:** 10.7759/cureus.89630

**Published:** 2025-08-08

**Authors:** Jim (Zhang Hao) Li, Timothy Kong, Emma M Dunne, Mitchell Liu, Jee Suk Chang, Tina Wanting Zhang, Matthew Chan, Ronan McDermott

**Affiliations:** 1 Division of Radiation Oncology and Developmental Radiotherapeutics, BC Cancer – Vancouver, Vancouver, CAN

**Keywords:** hypofractionation, metastatic neoplasm, palliative radiation therapy, radiation fractionation, radiation-induced toxicity, radiotherapy (rt)

## Abstract

Introduction

In select tumor sites, symptom palliation and local control can be improved through delivering higher biological equivalent doses (BED) of radiotherapy. However, not all patients are suitable candidates for stereotactic body radiation therapy (SBRT). The 30 Grays in five fractions (30/5) regimen is a conformal, hypofractionated regimen that offers a higher BED compared to conventional palliative radiotherapy. It adheres to the same organ-at-risk constraints as five-fraction SBRT. The planning and set-up processes are optimized to expedite treatment delivery compared to SBRT. This study aims to assess the oncologic and safety profiles of the 30/5 regimen.

Methods

Clinical data of patients who received the 30/5 regimen between October 2020 and August 2022 at our institution, the BC Cancer Vancouver Centre, were retrospectively reviewed. Local control (LC), distant metastasis-free survival (DMFS), progression-free survival (PFS), time to change in systemic therapy (CIS), and overall survival (OS) were calculated using the Kaplan-Meier method. Survival distributions between groups were compared using the log-rank test. Univariable and multivariable analyses were performed using Cox regression analysis to identify factors associated with the oncologic outcomes.

Results

Over 22 months, 77 patients received 92 courses of the 30/5 regimen. The median age was 68 years. The median tumor size treated was 11.4 cm^3^. The most common treatment locations were lung (34%), lymph nodes (21%), non-spine bone (18%), and spine (15%). At a median follow-up of 13.5 months, 29 deaths were observed. The 12-month LC was 66.1% (95% CI: 55.7-76.5%), and the 12-month DMFS, PFS, CIS, and OS were 35.9% (95% CI: 24.5-47.3%), 33.2% (95% CI: 22.0-44.4%), 59.2% (95% CI: 46.8-71.5%), and 72.5% (95% CI: 62.1-82.9%), respectively. The univariable analysis showed that radiosensitive and smaller tumors had better LC outcomes (p=0.005 and <0.001, respectively). These associations remained significant upon multivariable analysis too (p=0.035 and 0.026, respectively). No grade three or higher toxicity was reported.

Conclusion

The 30/5 regimen demonstrates good local control and favorable safety profile, particularly for small and radiosensitive tumors. Our findings suggest that 30/5 may be a viable alternative palliative regimen for patients who require high-dose radiotherapy, but are not suitable candidates for SBRT or prolonged interruptions in systemic therapy. In resource-limited settings, its hypofractionated approach may help conserve healthcare resources. Future studies are warranted to prospectively evaluate the impact of dosimetric distribution on oncologic outcomes.

## Introduction

Palliative radiotherapy has a long and successful track record of providing durable relief for patients with symptomatic metastatic disease. Higher biological equivalent doses of radiotherapy have been shown to improve symptom palliation and local control in select patient populations [1,2]. Stereotactic ablative radiotherapy (SBRT) is a technique for delivering high tumor-ablative doses and has been shown to be safe and beneficial in selected patients with a limited volume of metastases [3-5]. However, not all patients are candidates for SBRT, such as those with poor performance status, limited life expectancy, medical comorbidities prohibiting high-dose radiotherapy, or large tumor volumes that put them at an unacceptable risk of treatment-related toxicity [3,4]. In contrast, conventional palliative radiotherapy techniques deliver lower doses of radiation using fewer fields, but typically to larger volumes, with limited sparing of adjacent normal tissue from unnecessary low to intermediate doses [6,7].

The 30 Gy in five fractions regimen (30/5) in use at our institution stems from a modification of existing five-fraction SBRT regimens. Five-fraction SBRT regimens are considered the standard of care in many body sites requiring high-dose palliation [5,8,9]. The 30/5 regimen is delivered over five consecutive workdays, with six Gy per fraction. It is a conformal, homogeneous, hypofractionated regimen that uses volumetric modulated arc therapy (VMAT) to deliver high-dose palliative radiotherapy while adhering to the organ-at-risk (OAR) constraints for five-fraction SBRT. The hypofractionated approach allows for the delivery of higher radiation doses without increasing healthcare resource demands, while theoretically minimizing toxicity through respecting SBRT dose constraints.

From a radiobiological perspective, assuming an α/β of 10, 30/5 has an equivalent dose in two Gy per fraction (EQD2/10) of 40 Gy. This is comparable to other high-dose palliative regimens, such as 40 Gy in 15 fractions, which has an EQD2/10 of 42.2 Gy. However, as the 30/5 regimen has fewer fractions, this protocol may be more appropriate and better tolerated in those who are frailer or face logistical issues affecting treatment adherence. In addition, time off systemic treatment would be minimized for this group, making it a suitable option for patients who are dependent on systemic therapy. The 30/5 regimen offers a higher EQD2/10 compared to common low-dose palliative regimens, such as 30 Gy in 10 fractions (EQD2/10 of 33 Gy) or 20 Gy in five fractions (EQD2/10 of 23 Gy). Conversely, its EQD2/10 is lower than common SBRT regimens, such as 48 Gy in four fractions (EQD2/10 of 88 Gy), 60 Gy in eight fractions (EQD2/10 of 87.5 Gy), 35 Gy in five fractions (EQD2/10 of 49.6 Gy), and 54 Gy in three fractions (EQD2/10 of 126 Gy) [3]. Finally, from an administrative perspective, it frees up more treatment resources, thereby relieving strain on the healthcare system. In order to enhance patient safety and better understand its efficacy, this study endeavors to evaluate the oncologic and safety outcomes of patients receiving the 30/5 regimen.

## Materials and methods

Study design

Clinical and treatment data were retrospectively reviewed for all patients who received the 30/5 regimen between October 2020 and August 2022 at the BC Cancer Vancouver Centre, Vancouver, Canada, a comprehensive cancer center. Patients were offered the 30/5 regimen if they required high-dose palliative radiotherapy with a shorter fractionation regimen (such as to avoid additional interruptions in systemic therapy), and when they were not candidates for SBRT or clinical trials.

Exclusion criteria included tumor size ≥6 cm in maximum dimension (with the exception of non-spine bone metastases, provided that adjacent OAR constraints could be achieved), Eastern Cooperative Oncology Group (ECOG) performance status >3, life expectancy less than six months, and greater than two vertebral body levels involved. The final decision to treat was made in collaboration with the patient.

Data collection

Clinical data collected included patient information (age at diagnosis, age at treatment, biological sex, and ECOG performance status), disease characteristics (primary tumor site, pathologic grade, stage at diagnosis, and number of malignant lesions present at the time of treatment), and treatment history (site treated, size of lesion, date of treatment, systemic therapy courses, and other radiation therapy courses).

Outcome measures

The primary outcome was time to local failure (local control, LC). Secondary outcomes were distant metastasis-free survival (DMFS), progression-free survival (PFS), time to change in systemic therapy (CIS), and overall survival (OS). Local failure was defined as radiological or pathological evidence of tumor recurrence within the 50% isodose line, and LC was defined as the time from the completion of the 30/5 regimen to local failure. For LC, patients who did not experience local failure were censored at the date of last follow-up or death. DMFS was defined as time from completion of the 30/5 regimen to either radiologically or pathologically confirmed disease progression at a site outside the 50% isodose line. For DMFS, patients who did not experience distant progression were censored at the date of last follow-up or death. PFS was defined as time from completion of the 30/5 regimen to any site of disease progression, local or distant. For PFS, patients who did not experience disease progression were censored at the date of last follow-up or death. CIS was defined as time from completion of the 30/5 regimen to switching, initiating, or restarting systemic therapy. For CIS, patients who did not experience change in systemic therapy were censored at the date of last follow-up or death. OS was defined as time from completion of the 30/5 regimen until death from any cause. For OS, patients who were still alive at the date of last follow-up were censored at that date.

As this was a retrospective study, there were no standardized imaging schedules, and imaging was performed at the treating physician’s discretion. Toxicity was evaluated based on retrospective review of the clinician’s documentation. Acute toxicity was defined as occurring within 90 days of completion of the 30/5 regimen, and late toxicity was defined as occurring after 90 days of completion of the 30/5 regimen.

Data analysis

Data were analyzed on an intent-to-treat (ITT) basis. Patients who did not receive the full five fractions were also included in the chart review. Approval from the BC Cancer Agency Research Ethics Board (approval no: H22-02764) was obtained prior to initiation of the study.

Data were analyzed using IBM SPSS Statistics for Windows, Version 29 (Released 2022; IBM Corp., Armonk, New York, United States). The Kaplan-Meier method was used to calculate the primary and secondary outcomes. Survival distributions between groups were compared using the log-rank test. Univariable and multivariable Cox regression analyses were used to identify factors associated with the primary and secondary outcomes. Results were deemed statistically significant at p<0.05.

Factors assessed included age, biological sex, stage at initial diagnosis, performance status, radiosensitivity of the primary tumor, gross tumor volume (GTV) treated, maximum tumor dimension, number of malignant lesions, and receipt of systemic therapy prior to the 30/5 regimen. Radiosensitive primaries were defined as breast, prostate, lung, hematopoietic, gynecological, and head/neck malignancies, while radioresistant primaries were defined as gastrointestinal, renal cell, melanomatous, and sarcomatous malignancies [10,11]. An exception was made for adenoid cystic carcinoma, which was considered radioresistant [12]. Maximum tumor dimension of 3 cm was selected as a categorical cut off to examine the association between tumor size and LC, as multiple previous studies examining the efficacy of SBRT for various primary tumor sites have reported significant findings using a cutoff between 2.5 and 3.5 cm for their analyses [13,14]. Factors with a p-value <0.1 from the univariable analysis were entered into the multivariable analysis to identify independent predictors.

## Results

Between October 2020 and August 2022, 77 patients received 92 courses of the 30/5 regimen. Detailed patient and disease characteristics are summarized in Table 1.

**Table 1:** Demographic information and disease characteristics of patients who received the 30/5 regimen ECOG: Eastern Cooperative Oncology Group

Patient/Disease characteristic		Category	Number of patients (%)
Age at the time of receiving the 30/5 regimen		<40	5 (6)
		40-50	5 (6)
		50-60	7 (9)
		60-70	23 (30)
		70-80	21 (27)
		80-90	12 (16)
		>90	4 (5)
Biological sex		Male	41 (53)
		Female	36 (47)
ECOG Performance Status		0	14 (18)
		1	42 (55)
		2	17 (22)
		3	4 (5)
Primary tumor site	Radiosensitive	Lung	34 (44)
		Breast	8 (10)
		Prostate	4 (5)
		Head and neck	3 (4)
		Gynecological	2 (3)
	Radioresistant	Gastrointestinal	15 (19)
		Renal cell	4 (5)
		Melanoma	3 (4)
		Sarcoma	3 (4)
		Adenoid cystic	1 (1)
Number of malignant lesions at the time of receiving the 30/5 regimen		1	20 (26)
		2	14 (18)
		3	13 (17)
		4	5 (6)
		5 or more	25 (32)

The median age at the time of diagnosis was 63 years (range: 27-93 years). The median age at the time of receiving the 30/5 regimen was 68 (range: 37-93 years). Younger age was positively correlated with a more advanced stage at diagnosis (Pearson correlation coefficient: 0.363, p<0.002). The median GTV treated was 11.4 cm^3^ (range: 0.3-210.9 cm^3^). The median greatest tumor dimension was 2.4 cm (interquartile range: 1.5-3.8 cm). Detailed data regarding systemic and other radiation treatment history can be found in Table 2.

**Table 2:** Treatment information of patients who received the 30/5 regimen

Treatment parameter		Number of lesions (%)
Location treated with radiotherapy	Lung	31 (34)
	Lymph nodes	19 (21)
	Spine	14 (15)
	Non-spine bone	17 (18)
	Other sites	11 (12)
		Number of patients (%)
Systemic therapy	Initiated before the 30/5 regimen and changed/restarted after completion of the 30/5 regimen	25 (32)
	Initiated before the 30/5 regimen, no change/restart after completion of the 30/5 regimen	23 (30)
	Initiated after the completion of the 30/5 regimen	7 (9)
	Never received systemic therapy	22 (29)
Other radiotherapy (to any site)	Received before and after the 30/5 regimen	12 (16)
	Received only before the 30/5 regimen	31 (40)
	Received only after the 30/5 regimen	13 (17)
	Never received other radiotherapy	21 (27)

At a median follow-up of 13.5 months, 29 deaths occurred. There were five acute grade two toxicities. These included two cases of cough requiring narcotic antitussives, one case of pneumonitis requiring steroids, one case of nausea requiring ondansetron, and one case of tumor pain flare. One patient had late grade two pneumonitis seven months after receiving the 30/5 regimen, which required treatment with steroids. No grade three or higher toxicity was reported. Of the 77 patients treated, only one did not complete the course of the 30/5 regimen due to worsening frailty, thought to be secondary to progressive encephalitis, unrelated to the radiotherapy treatment.

Using the Kaplan-Meier survival analysis, the mean LC for all 92 treatment courses was 21.0 months (95% confidence interval [CI]: 18.2-23.7 months). Median LC could not be calculated due to a lack of events. There were 34 cases of local failure (37% of all treatment courses). Median DMFS for all 77 patients was seven months (95% CI: 3.9-10.0 months), and 12-month DMFS was 35.9% (95% CI: 24.5-47.3%), with 50 cases of distant failure (65% of all patients). Median PFS was 6.8 months (95% CI: 4.1-9.5 months), and 12-month PFS was 33.2% (95% CI: 22.0-44.4%), with 53 cases of either local or distant disease progression (69% of all patients). Median CIS was 15.8 months (95% CI: 9.0-22.5 months), and 12-month CIS was 59.2% (95% CI: 46.8-71.5%), with 32 cases of initiation, change, or restart of systemic therapy (42% of all patients). Median OS was 25.6 months (95% CI: 20.5-30.7 months), and 12-month OS was 72.5% (95% CI: 62.1-82.9%).

**Figure 1:**
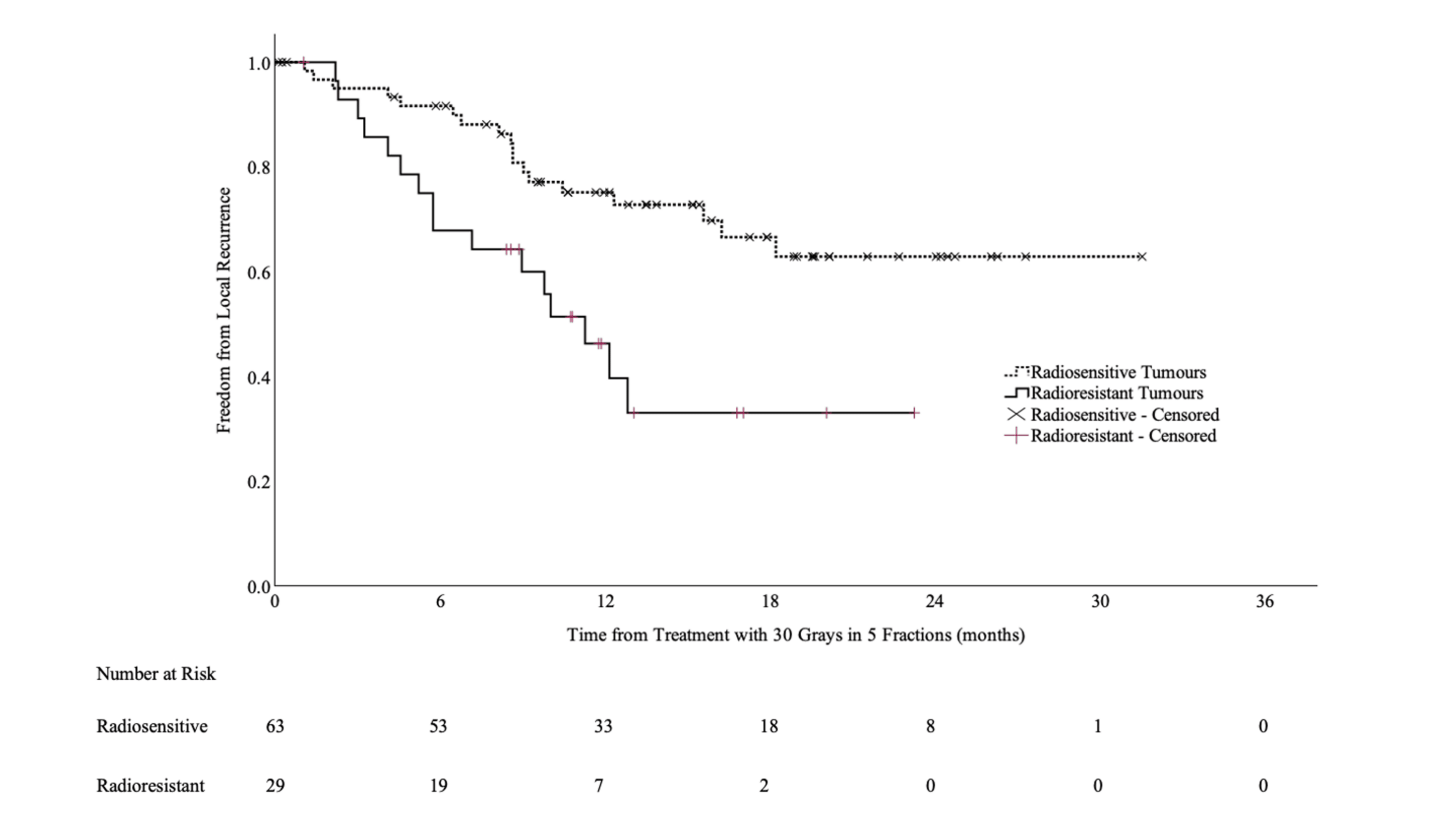
Kaplan-Meier local control survival curves for radiosensitive vs radioresistant primary tumors

The LC for all treatment courses at 12 months was 66.1% (95% CI: 55.7-76.5%). The 12-month LC for radiosensitive and radioresistant primaries was 75.2% (95% CI: 63.8-86.6%) and 46.3% (95% CI: 26.5-66.1%, p<0.005), respectively (Figure 1).

The 12-month LC for treated lesions <3.0 cm in maximum dimension, was 76.8% (95% CI: 65.6-88.0%). This was statistically higher than that of lesions ≥3 cm, which had a 12-month LC of 44.4% (95% CI: 25.2-63.6%; p<0.01). The 12-month LC for treated lesions located in the lung, lymph nodes, spine, and non-spine bone were 56.2% (95% CI: 46.6-65.8%), 77.8% (95% CI: 68.0-87.6%), 81.5% (95% CI: 69.6-93.4%), and 62.7% (95% CI: 50.5-74.9%), respectively.

On univariable analysis, tumor radioresistance (hazard ratio [HR] 2.631, 95% CI: 1.329-5.206; p=0.005), maximum dimension ≥3.0 cm (HR 1.336, 95% CI: 1.082-1.651, p=0.007), and higher GTV (HR 1.013, 95% CI: 1.006-1.020; p<0.001) were associated with inferior LC. Age (p=0.118), biological sex (p=0.581), stage at diagnosis (p=0.457), ECOG at the time of receiving the 30/5 regimen (p=0.357), having five or more malignant lesions at the time of receiving the 30/5 regimen (p=0.106), and prior systemic therapy (p=0.717) were not statistically significant for LC. On multivariable analysis, only radioresistance was significantly associated with inferior LC (Table 3).

**Table 3:** Univariable and multivariable analysis of local control N/A: Not applicable; ECOG: Eastern Cooperative Oncology Group; GTV: Gross tumor volume

Variable	Univariable (Cox regression)		Multivariable (Cox regression)	
	P value	Hazard Ratio (95% CI)	P value	Hazard Ratio (95% CI)
Age	0.118	0.983 (0.961-1.004)	N/A	N/A
Biological sex	0.581	1.216 (0.607-2.436)	N/A	N/A
Primary radiosensitivity	0.005	2.631 (1.329-5.206)	0.046	2.067 (1.012-4.220)
Stage at diagnosis	0.457	1.124 (0.826-1.528)	N/A	N/A
ECOG Performance Status at the time of receiving the 30/5 regimen	0.357	1.235 (0.788-1.935)	N/A	N/A
Number of malignant lesions (≥5)	0.106	1.744 (0.889-3.423)	N/A	N/A
Prior systemic therapy	0.717	1.147 (0.547-2.405)	N/A	N/A
Tumor diameter (≥3.0 cm)	0.007	1.336 (1.082-1.651)	0.710	1.067 (0.760-1.497)
GTV radiated	<0.001	1.013 (1.006-1.020)	0.107	1.010 (0.998-1.022)

Tables 4-7 summarize the univariable and multivariable analyses on factors affecting the secondary outcomes (DMFS, PFS, CIS, and OS), respectively.

**Table 4:** Univariable and multivariable analysis of distant metastasis-free survival N/A: Not applicable; ECOG: Eastern Cooperative Oncology Group; GTV: Gross tumor volume

Variable	Univariable (Cox regression)		Multivariable (Cox regression)	
	P value	Hazard Ratio (95% CI)	P value	Hazard Ratio (95% CI)
Age	<0.001	0.948 (0.928-0.970)	0.002	0.961 (0.936-0.985)
Biological sex	0.854	0.949 (0.544-1.654)	N/A	N/A
Primary radiosensitivity	<0.001	2.901 (1.624-5.180)	0.015	2.233 (1.170-4.263)
Stage at diagnosis	<0.001	1.818 (1.358-2.433)	0.011	1.588 (1.113-2.265)
ECOG Performance Status at the time of receiving the 30/5 regimen	0.192	1.268 (0.888-1.810)	N/A	N/A
Number of malignant lesions (≥5)	<0.001	3.125 (1.748-5.584)	0.251	1.451 (0.768-2.742)
Prior systemic therapy	0.114	1.618 (0.891-2.941)	N/A	N/A
Tumor diameter (≥3.0 cm)	0.172	1.477 (0.844-2.587)	N/A	N/A
GTV radiated	0.010	1.009 (1.002-1.016)	0.046	1.009 (1.000-1.017)

**Table 5:** Univariable and multivariable analysis of progression-free survival N/A: Not applicable; ECOG: Eastern Cooperative Oncology Group; GTV: Gross tumor volume

Variable	Univariable (Cox regression)		Multivariable (Cox regression)	
	P value	Hazard Ratio (95% CI)	P value	Hazard Ratio (95% CI)
Age	<0.001	0.961 (0.940-0.982)	0.020	0.971 (0.948-0.995)
Biological sex	0.989	0.996 (0.580-1.711)	N/A	N/A
Primary radiosensitivity	<0.001	2.648 (1.502-4.671)	0.027	2.057 (1.087-3.893)
Stage at diagnosis	<0.01	1.600 (1.225-2.091)	0.035	1.409 (1.025-1.937)
ECOG Performance Status at the time of receiving the 30/5 regimen	0.101	1.328 (0.946-1.864)	N/A	N/A
Number of malignant lesions (≥5)	<0.001	3.125 (1.748-5.584)	0.382	1.325 (0.706-2.487)
Prior systemic therapy	0.407	1.271 (0.721-2.242)	N/A	N/A
Tumor diameter (≥3.0 cm)	0.047	1.750 (1.008-3.038)	0.395	1.320 (0.696-2.502)
GTV radiated	0.002	1.010 (1.004-1.017)	0.118	1.007 (0.998-1.016)

**Table 6:** Univariable and multivariable analysis of time to change in systemic therapy N/A: Not applicable; ECOG: Eastern Cooperative Oncology Group; GTV: Gross tumor volume

Variable	Univariable (Cox regression)		Multivariable (Cox regression)	
	P value	Hazard Ratio (95% CI)	P value	Hazard Ratio (95% CI)
Age	<0.001	0.962 (0.940-0.984)	0.086	0.973 (0.944-1.004)
Biological sex	0.505	0.788 (0.392-1.586)	N/A	N/A
Primary radiosensitivity	<0.001	3.413 (1.673-6.962)	0.003	3.134 (1.456-6.746)
Stage at diagnosis	0.045	1.409 (1.008-1.970)	0.690	1.088 (0.719-1.648)
ECOG Performance Status at the time of receiving the 30/5 regimen	0.442	0.819 (0.491-1.363)	N/A	N/A
Number of malignant lesions (≥5)	0.016	2.371 (1.178-4.774)	0.926	0.960 (0.402-2.294)
Prior systemic therapy	0.084	2.099 (0.906-4.858)	0.442	1.466 (0.553-3.889)
Tumor diameter (≥3.0 cm)	0.260	1.497 (0.742-3.020)	N/A	N/A
GTV radiated	0.117	1.007 (0.998-1.015)	N/A	N/A

**Table 7:** Univariable and multivariable analysis of overall survival N/A: Not applicable; ECOG: Eastern Cooperative Oncology Group; GTV: Gross tumor volume

Variable	Univariable (Cox regression)		Multivariable (Cox regression)	
	P value	Hazard Ratio (95% CI)	P value	Hazard Ratio (95% CI)
Age	0.888	1.002 (0.976-1.028)	N/A	N/A
Biological sex	0.579	0.579 (0.273-1.228)	N/A	N/A
Primary radiosensitivity	0.003	3.115 (1.457-6.658)	0.010	2.782 (1.276-6.066)
Stage at diagnosis	0.629	0.922 (0.663-1.282)	N/A	N/A
ECOG Performance Status at 30/5	0.002	2.225 (1.344-3.685)	0.005	2.228 (1.277-3.889)
Number of malignant lesions (≥5)	0.335	1.442 (0.686-3.033)	N/A	N/A
Prior systemic therapy	0.517	0.777 (0.362-1.668)	N/A	N/A
Tumor diameter (≥ 3.0 cm)	0.086	1.929 (0.912-4.079)	0.689	0.818 (0.306-2.190)
GTV radiated	0.003	1.012 (1.004-1.020)	0.109	1.008 (0.998-1.018)

## Discussion

The retrospective cohort of 77 patients who underwent the 30/5 regimen was heterogeneous in age, performance status, primary tumor histology, and tumor burden. Additionally, the size and location of the radiated lesions were also quite diverse. The 30/5 patient cohort was specifically selected, as these patients were not ideal candidates for SBRT due to concerns such as shorter disease-free interval, polymetastatic disease, large tumors with limiting constraints due to adjacent OAR, or comorbidities that precluded stringent SBRT setup or immobilization. However, these patients could still benefit from more durable LC than conventional palliative radiotherapy, as they had reasonable performance status, a life expectancy greater than six months, and relatively stable disease elsewhere.

Since the 30/5 regimen is designed to be an intermediate between conventional palliative radiotherapy and SBRT, its LC would be expected to fall somewhere in between. However, without a control group or an SBRT cohort embedded within our study, we are limited to comparing our results to those of previously published studies. We acknowledge that cross-study comparisons must be interpreted with caution, as the patient cohort receiving the 30/5 regimen represents a distinct population from that of SBRT or conventional palliative radiotherapy. The stereotactic ablative radiotherapy (SABR) for up to five oligometastases (SABR-5) study has demonstrated that LC for SBRT at 12 months was 93% (95% CI: 91-95) [15]. Although this is higher compared to what we found with the 30/5 regimen, 66.1% (95% CI: 60.8-71.4%), it should be noted that SABR-5 had more stringent inclusion and exclusion criteria regarding patient performance status, lesion size, and number of oligometastases, whereas these criteria were less strict for the 30/5 regimen. When we examined the LC of the 30/5 regimen by primary radiosensitivity and lesion size, we found that radiosensitive tumors had 12-month LC rates of 75.2% (95% CI: 69.4-81.0%), and tumors with a maximal dimension <3.0 cm had a 12-month LC of 76.8% (95% CI: 71.1-82.5%). While these values are still lower than what is established for SBRT, they are higher compared to conventional palliative radiotherapy, which typically has a 12-month LC in the range of 40-70% [2,16,17]. A recent study comparing spine SBRT to conventional palliative radiotherapy specifically noted a 12-month local failure rate of 6.1% (95% CI: 2.5-12.1%) for SBRT versus 28.4% (95% CI: 21.3-35.9%) for conventional external beam radiotherapy [17]. In our study, the 30/5 regimen given to spine lesions had a 12-month LC rate of 81.5% (95% CI: 69.6-93.4%), corresponding to a local failure rate of approximately 18.5%, which is expected for an intermediate-dose regimen based on the prior study’s findings. This is unsurprising, as SBRT delivers a higher biologically equivalent dose with more hotspots in the dose distribution compared to the homogeneous dose distribution of the 30/5 regimen, and patients receiving SBRT typically have better performance status and would thus be eligible for more systemic therapy options, which could also prolong LC.

Univariable analysis of factors affecting LC showed that radiosensitive primaries (p=0.005), smaller GTV (p<0.001), and maximum tumor dimension <3.0 cm (p=0.007) were associated with significantly better LC. These results are expected, as larger tumor sizes may limit radiotherapy coverage in favor of respecting dose constraints. Additionally, larger tumors may harbor greater hypoxic regions, which in turn reduce radiosensitivity [18]. The inverse relationship between tumor size and LC has been demonstrated in previous SBRT studies [13,14,19]. Upon multivariable analysis, only radiosensitivity was statistically significant for LC (p=0.046; Table 3), indicating that the primary tumor histology should be considered when selecting patients for the 30/5 regimen.

On univariable analysis, tumor radioresistance (p=0.003), worse performance status (p=0.002), and greater GTV volume (p=0.003) were correlated with worse OS outcomes (Table 7). The association between OS and radioresistance (p=0.010), and performance status (p=0.005) remained significant on multivariable analysis. While it is expected that poorer performance status is predictive of death, it is interesting to note that radioresistance and GTV volume were associated with OS. We could hypothesize that, because they are shown to be correlated with better LC, there may be some synergistic effect, whereby superior LC of one malignant lesion may have a protective effect on OS, an observation seen in select patient populations with prostate cancer, as evidenced by the Systemic Therapy for Advancing or Metastatic Prostate Cancer: Evaluation of Drug Efficacy (STAMPEDE) trial [20,21].

Overall, the 30/5 regimen was well tolerated, with only five acute grade two toxicities, one late grade two toxicity, and no grade three or higher toxicities reported. In total, there were six cases of grade two toxicity out of 92 courses of the 30/5 regimen (6.5% of all treatment courses). With the caveat of cross-study comparisons, this is lower than what is reported in the literature for SBRT, which typically ranges between 10-30% [22,23]. Literature values for toxicity associated with conventional palliative radiotherapy vary widely depending on the era, dosage, and location radiated, but reported values tend to range between 10-40% [24,25], again higher than what we observed with the 30/5 regimen. The 30/5 protocol was designed to respect the normal tissue constraints for the five-fraction SBRT, give higher prioritization to OARs compared to conventional palliative radiotherapy, and deliver a lower dose compared to SBRT. These factors may have contributed to the favorable toxicity profile of this regimen.

From a resource and convenience perspective, the 30/5 regimen presents an alternative fractionation schedule that offers durable local control without the need for a multi-week treatment course. Our center has a vast geographical catchment, with some patients travelling over a 1000 kilometers to receive radiotherapy. In settings where access to care poses a challenge, the benefits of hypofractionation include better treatment compliance, improved access to care, decreased costs for the healthcare system, and reduced financial toxicity for patients [26,27]. Additionally, previous studies have shown a substantial reduction in radiotherapy-related greenhouse gas emissions by using hypofractionation regimens when clinically appropriate [28,29]. In the palliative context, an estimated 31% to 56% of greenhouse gas emissions could be eliminated through hypofractionation [29].

Limitations to our study include the fact that the patient population receiving the 30/5 regimen was distinct from those receiving SBRT and conventional radiotherapy, so extra caution must be applied when making cross-study comparisons. Because this is a single-institution study, introducing the protocol to other centers and performing quality assurance studies would be paramount to ensuring that our results are indeed generalizable to other settings. Future research efforts could be directed towards conducting multicenter randomized controlled trials or prospective studies to better compare the 30/5 regimen with SBRT and conventional palliative radiotherapy.

## Conclusions

The 30/5 regimen was generally well-tolerated. It achieved an acceptable LC of approximately 75% at 12 months for malignant lesions that were radiosensitive, less than 3.0 cm in maximum dimension, or located in the lymph nodes or spine. Our findings suggest that this regimen is a viable alternative for select patients who are not ideal candidates for SBRT but require durable palliative radiotherapy with minimal disruption to their systemic therapy. In resource-limited settings, its hypofractionated approach may also help conserve healthcare resources. These results may inform future prospective studies aimed at further characterizing the safety and efficacy profile of the 30/5 regimen. Additional research is warranted to assess the impact of dosimetric distribution on oncologic outcomes.
